# Integrated Resource Management for Fog Networks

**DOI:** 10.3390/s22062404

**Published:** 2022-03-21

**Authors:** Jui-Pin Yang, Hui-Kai Su

**Affiliations:** 1 Department of Information Technology and Communication, Shih-Chien University, Kaohsiung 845, Taiwan; 2 Department of Electrical Engineering, National Formosa University, Yunlin 632, Taiwan; hksu@nfu.edu.tw

**Keywords:** resource management, fog network, energy perception, service level agreement, load balancing, hotspot offload

## Abstract

In this paper, we consider integrated resource management for fog networks inclusive of intelligent energy perception, service level agreement (SLA) planning and replication-based hotspot offload (RHO). In the beginning, we propose an intelligent energy perception scheme which dynamically classifies the fog nodes into a hot set, a warm set or a cold set, based on their load conditions. The fog nodes in the hot set are responsible for a quality of service (QoS) guarantee and the fog nodes in the cold set are maintained at a low-energy state to save energy consumption. Moreover, the fog nodes in the warm set are used to balance the QoS guarantee and energy consumption. Secondly, we propose an SLA mapping scheme which effectively identifies the SLA elements with the same semantics. Finally, we propose a replication-based load-balancing scheme, namely RHO. The RHO can leverage the skewed access pattern caused by the hotspot services. In addition, it greatly reduces communication overheads because the load conditions are updated only when the load variations exceed a specific threshold. Finally, we use computer simulations to compare the performance of the RHO with other schemes under a variety of load conditions. In a word, we propose a comprehensive and feasible solution that contributes to the integrated resource management of fog networks.

## 1. Introduction

In the paper, we propose integrated resource management for fog networks including load balancing, energy perception and SLA planning. The load balancing is the most important issue in integrated resource management because it plays a key role in the overall performance of the fog networks. The main functionality of load balancing schemes is to deal with bursty and unbalanced load conditions in fog networks. Unfortunately, load-balancing schemes that achieve excellent fairness are generally too complicated to implement. Apparently, the fog nodes that contain hotspot services have relatively heavier loads. Many replication-based load-balancing schemes have been proposed, which could be roughly divided into four categories. In the first category, all stored data are replicated to all fog nodes in a fog network regardless of their load conditions [1,2,3]. This strategy is easy to implement. However, it consumes a lot of resources inclusive of storage space and network bandwidth whenever data replication happens. The second category only stores a replica of stored data and consequently it has the highest storage utilization. However, it can only work well under very limited load conditions because there are no additional replicas. The third category keeps a pre-defined number of replicas for stored data and hence the number of replicas cannot be adjusted to cope with load variations. Furthermore, it needs specific request scheduling algorithms to efficiently distribute the arriving requests to adequate fog nodes, such as round robin [4]. The fourth category dynamically constructs an adequate number of replicas according to load conditions [5,6,7,8]. In order to deal with fairness, simplicity and resource efficiency, we propose a novel replication-based load-balancing scheme that works well under bursty and unbalanced load conditions. More importantly, it only requires small numbers of replicas. As a result, it is very suitable to be deployed for fog networks whose resources are limited.

A high locality of reference exists especially for extremely large file systems [9]. For instance, some files possess extremely unbalanced access behaviors as compared with other files. Similarly, some data are extremely popular and therefore they have relatively higher access frequencies [10]. To efficiently reduce energy consumption, the main idea is to dynamically classify the fog nodes into hot set, warm set or cold set. In the hot set, fog nodes maintain the replicas of hotspot services, which continuously work in active state for achieving QoS requirements. In addition, the fog nodes that belong to the cold set are used to maintain the replicas of the non-hotspot service. They also work in inactive state. The warm set is a temporary state between active state and inactive state. The fog nodes in the warm set dynamically switch to active state or inactive state depending on overall system situations. In other words, they maintain the replicas of hotspot services or non-hotspot services on demand. Apparently, our proposal is capable of load balancing and intelligent energy perception. Finally, we incorporate SLA planning and hence users can systematically represent their QoS requirements that contribute to constructing SLA contracts. We conclude the contributions of this study as follows:

(1) We propose an intelligent energy perception scheme capable of efficient energy management. The fog nodes are dynamically classified into a hot set, warm set or cold set in terms of load conditions. The fog nodes in the hot set are responsible for QoS guarantee and the fog nodes in the cold set are used to reduce energy consumption. Additionally, the fog nodes that belong to the warm set are used to deal with sudden load conditions in time, while balancing energy consumption.
(2) We propose an SLA mapping scheme for SLA planning, which systematically describes the relationship between resource metrics and SLA parameters. Moreover, this scheme can identify the SLA elements with the same semantics.
(3) We propose a replication-based hotspot offload (RHO) scheme which achieves approximately perfect fairness under different load conditions. In addition, it is suitable to be deployed in fog networks whose resources are limited.

The remainder of the paper is organized as follows; in Section 2, we present the related work. Section 3 explains the details of the integrated resource management inclusive of intelligent energy perception, SLA planning and replication-based hotspot offload. Section 4 compares the fairness of the RHO scheme with other load-balancing schemes under various load conditions. Finally, we conclude the contributions and future work in Section 5.

## 2. Related Work

In this section, we review previous studies associated with integrated resource management. First of all, we introduce the load-balancing schemes. A block placement strategy that determines the best data nodes achieves real-time responses in the Hadoop environment [11]. This strategy requires preliminary replicas so it improves data synchronization. However, it has poor fairness. One study investigated the effects of job scheduling and data replication [12]. In order to efficiently manage the number of replicas, an auction protocol that combines an economic model was proposed. However, this proposal was evaluated under European data grid environments. Two ways are generally used to handle the arriving requests of replication-based load-balancing schemes and they both are classified into centralized control and distributed control [13,14,15]. In centralized control, the fog controller must select the best fog nodes for processing the arrival requests. In addition, the centralized control needs to collect real-time fog node information and therefore it results in huge additional communication overheads and larger latency. Moreover, all arriving requests are handled by the fog controller which might become the performance bottleneck. In distributed control, arriving requests are mainly handled by corresponding master fog nodes. Consequently, it greatly reduces communication overheads and gets rid of the performance bottleneck. Therefore, we take distributed control into account. Several load-balancing schemes require a prior knowledge used to efficiently deal with arriving requests [16]. Comparatively speaking, the other load-balancing schemes do not require any prior knowledge but they rely on other specific information, for instance, the system status or the amount of awaiting requests in each server. A solution that adopts a low-level load balancer works at the network level in the open system interconnection model [17]. When a request arrives, it is forwarded to a back-end server. Next, the header of the request is modified according to a mapping table. However, this scheme has non-linear speedup proportional to the number of servers.

A load-balancing algorithm was proposed to deal with the conditions where the back-end servers should be familiar with the front-end servers [18]. As a result, it cannot be applied to conditions where the end-users can directly access back-end servers. Similarly, a dynamic load-balancing scheme that utilizes dynamic DNS updates and a round-robin mechanism was proposed [19]. The scheme can add or remove a server from the DNS list according to load variations. Accordingly, it decreases response time because the overloaded servers can be dynamically removed from the DNS list. A scheduling algorithm that depends on resource usages such as CPU, memory and bandwidth accordingly was also proposed. One scheme reduces scheduling overheads by creating several replicas of each job. Therefore, the requests could be effectively dispatched to adequate replicas [20]. When a replica reaches the head of the queue at one of the servers, the other servers that have the same replica of the job are removed from corresponding queues. Although it is useful to improve queuing overheads, inter-server communication still could lead to performance degradation such as high propagation delay. MapReduce is a distributed programming model and therefore it is very suitable for the development of large-scale data-parallel applications [21]. A load-balancing algorithm that considers node performance is applied to address the problem of data assignment. After the map phase, the execution time can be balanced for the reduce tasks [22]. 

In order to enhance system resource utilizations and constrain the frequencies of message exchange, a load-balancing strategy that incorporates an information exchange policy consisting of a random walk and a decentralized nature was proposed [23]. This strategy exchanges information using random packets so that each node keeps up-to-date knowledge of the other nodes. Furthermore, two message replication strategies are used to improve the efficiency and scalability of unstructured P2P networks while maintaining query performance [24]. First of all, distance-based message replication strategy replicates query messages among different topological regions. Next, landmark-based strategy optimizes query processing because both the topology and physical proximity of peers are considered. A dynamic replica technique utilizes acceleration to resolve reliability and latency for cloud storage [25]. This technique can identify hotspot data in the next period based on acceleration information. Accordingly, it selects the best node and then creates a new replica. One strategy uses the session time to explore time-related replication that get rid of bursty failures under P2P-based storage systems [26]. Furthermore, it provides sufficient time to replace the lost replica using the primary replica. A dynamic replication strategy was proposed to reduce delay because it considers access costs [27]. The strategy not only enhances load balancing but also improves data availability. However, it is too complicated to implement. 

Several energy perception schemes enable the servers to work at low energy states. Therefore, the servers decrease energy consumption of high-performance applications with virtualization [28]. A system alleviates the impact of time-varying loads in fog networks because it can dynamically control the on/off states of the fog nodes [29]. The system modifies reconfiguration decisions by considering the overall load impact on the system, energy and performance. As a result, several adjustment policies were proposed to modify current configurations. With various combinations of dynamic voltage scaling and node states, the policies decrease the aggregate energy consumption within a server cluster when the overall workload is low [30]. A runtime system utilizes energy management that supports system-wide, application-independent, dynamic voltage and frequency scaling in a generic energy-aware cluster [31]. The system achieves 20% energy saving based on NAS parallel benchmark. In addition, it can constrain the performance degradation of most applications under user-specified limits. A run-time scheduling algorithm was proposed to improve energy consumption in cluster systems [32]. An energy-aware algorithm possesses energy reduction accompanied with minimal impact on performance by adjusting the settings of voltage and frequency [33]. In the past, load balancing and energy perception were studied separately and therefore in the idea that we consider, both load balancing and energy perception are novel and useful. 

Finally, we review the work related to the SLA planning. The transfer functions are used to represent the relationships between resource metrics and QoS attributes in web services [34]. Furthermore, it continuously monitors and evaluates the states of QoS attributes so as to guarantee QoS requirements. On the other hand, it lacks monitoring on resource metrics, that results in infeasibility. LoM2HiS architecture manages the relationships between resource metrics and SLA parameters [35]. The architecture monitors the resource metrics and SLA parameters so that SLA violations can be detected. Furthermore, a prevention policy is used to deal with SLA violations. However, data inconsistencies may occur because many replicas are needed. More importantly, single point of failure is an unavoidable problem herein. The web service level agreement (WSLA) is an XML-based agreement [36]. In addition, the WSLA can be extended to establish new metrics based on current metrics. In other words, it can be used to implement multiple SLA parameters. The WS-Agreement was developed for grid computing [37]. The WS-Agreement is an XML-based agreement, which is generally used to represent the non-functional attributes of an SLA contract in web services. We found that the WSLA is relatively suitable for fog networks.

SLA management was proposed to multiple software architectures, including the operation layer, application layer, middle layer and basic layer [38]. In addition, a QoS model that associates with different SLA layers was proposed. However, it lacks feasibility because it only demonstrates a multilayer abstract software architecture. The SLA guarantee and penalty are two common components in SLA planning. For instance, the maximum and minimum thresholds should be set up in order to guarantee the SLA requirements. Once the SLA is violated, some penalty policies should be adopted for the service providers. In general, the users would like to satisfy their service level objectives with minimum cost. Inversely, service providers expect to get maximum revenue with minimum resources. An agent system reaches SLA negotiation based on auction policy [39]. SLA negotiation consists of three phases inclusive of negotiation media, auction selection and SLA configuration. Once the SLA negotiation is completed, it means that an SLA contact has been established. The auction procedures are too complicated to rapidly complete SLA negotiation. Therefore, additional time is necessary. The SLA negotiation can use ontology to realize the automatic SLA matches and then select the adequate service providers [40,41,42]. Apparently, the SLA mapping is a key component because it is mainly responsible for bridging the differences of SLAs [43,44]. In this study, we propose a novel SLA mapping mechanism which is the core of the SLA planning. Furthermore, the proposed mechanism is capable of rapidly identifying the SLA elements with the same semantics. 

## 3. Integrated Resource Management

**Figure 1:**
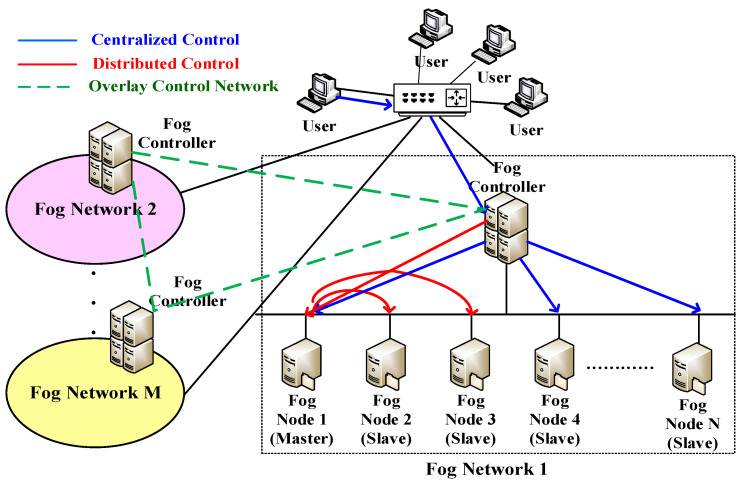
System architecture of fog networks.

A system architecture that consists of M fog networks is depicted in Figure 1. In addition, a fog network consists of different numbers of fog nodes, for instance, fog network 1 consists of N fog nodes. Moreover, there is at least one fog controller in each fog network. The number of fog controllers is proportional to the scales of the fog networks. The fog controllers are responsible for managing fog nodes in corresponding fog networks. Besides, they communicate with other fog controllers regarding resource sharing among different fog networks. There are two ways to deal with arriving requests which include centralized control and distributed control. In centralized control, all arriving requests are completely handled by the fog controller. It means that the fog controller has to determine the best fog node to process the arriving requests. In addition, the fog controller uses a scheduling algorithm whose functionality is to schedule the arriving requests to fog nodes with the required services according to specific performance metrics, such as load conditions, resource usages and latency. Undoubtedly, centralized control is easy to implement, but it imposes a heavy burden on fog controllers. In other words, fog controllers may become the performance bottleneck. Besides, it may lead to high communication overheads between the fog controllers and fog nodes. In distributed control, the fog controller looks up the master fog nodes with the required service and then it directly forwards the arriving requests to the selected master fog node. Then, the fog controller is unnecessary to handle the following processing. The master fog node maintains the original service, which dispatches arriving requests to other fog nodes (slave fog nodes) with the replica of required service based on load conditions. A replica means a duplicate of the same service. In other words, the master fog node is responsible for the partial tasks of the fog controller so that it can relieve the load burden of the fog controller. Moreover, the master fog node may play the role of a slave fog node, depending on the originality of services. The access patterns of hotspot service usually have short-term and unpredictable characteristics. Therefore, load imbalance frequently happens. With the replicas, the master fog node can effectively distribute arriving requests to other slave fog nodes, thus improving overall load balancing. Accordingly, we consider the distributed control in this study. 

**Figure 2:**
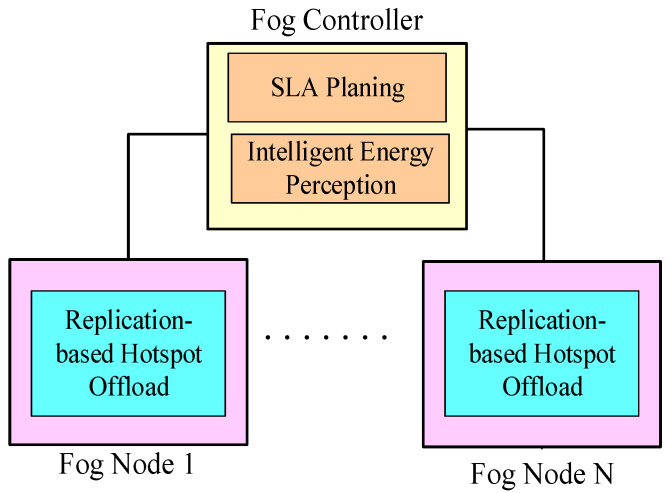
The framework of integrated resource management.

To achieve efficient resource management among fog networks, the fog controllers are responsible for configuring an overlay control network that enhances resource sharing. The consistency of the overlay control network depends on the physical proximity of the fog networks. In the overlay control network, the fog controllers dynamically update their system information to others whenever the variations of monitored resources exceed pre-defined thresholds. For instance, when the overall load of a specific fog network turns into a high value, the corresponding fog controller will communicate with other fog controllers via the overlay control network. Next, the fog controllers will be in charge of looking up the fog node with the lowest load and sending the relative information of the selected fog node back to the corresponding fog controller. Next, the fog controller selects the best fog node according to the received information if new replicas are needed. Once the hotspot effect is eliminated, the replica can be deleted on demand. As a result, the load variations can be distributed to different fog networks and therefore it improves overall load balancing. In order to simplify simulations and analysis, we consider one fog network in this study. However, it is applicable to multiple fog networks. The framework of the integrated resource management is described in Figure 2, which is composed of three components including replication-based hotspot offload (RHO), intelligent energy perception and SLA planning. The fog controllers contain two components including intelligent energy perception and SLA planning. The fog nodes contain one component, namely RHO.

**Figure 3:**
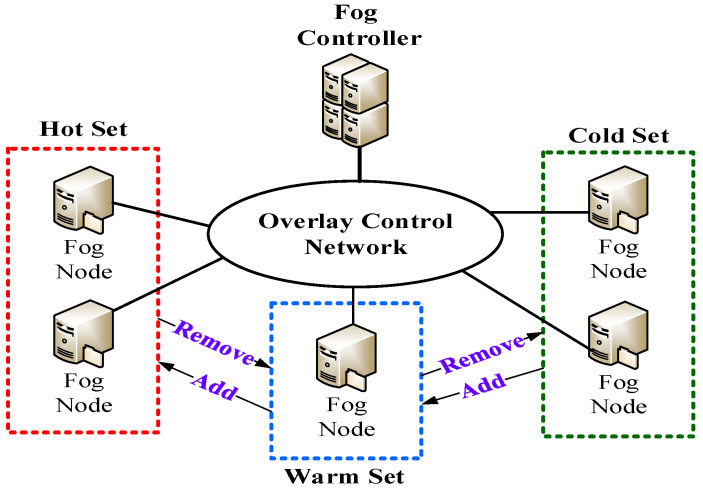
Architecture of intelligent energy perception.

Figure 3 is the architecture of intelligent energy perception where the fog controllers are responsible for power management and the fog nodes are responsible for physical power handling. In the overlay control network, all fog nodes in different fog networks are dynamically classified into a hot set, warm set or cold set based on load conditions or a pre-defined number of fog nodes in each set. The hotspot services generally maintain multiple replicas which are distributed to the fog nodes belonging to the hot set. In addition, the seldomly accessed replicas are stored in the fog nodes belonging to the cold set. According to the 80/20 rule, the hotspot services occupy a small proportion of total services in the overlay control network. Consequently, the number of fog nodes in the hot set is normally smaller than that in the cold set and warm set. In order to rapidly recover the request processing, some fog nodes are selected and they belong to the warm set. Apparently, the fog nodes can be automatically added/removed from the overlay control network. When the fog nodes belong to the hot set, they support high performance with high energy state. When the fog nodes belong to the warm set, they maintain at medium energy state and hence they can recover in time. Finally, the fog nodes that belong to the cold set maintain at low energy state and therefore energy consumption can be reduced. Accordingly, it achieves intelligent energy perception. It is easy to extend the RHO to achieve intelligent energy perception while keeping excellent load balancing. When the RHO needs to create a replica of a required service, the new replica will be sequentially allocated to one of the fog nodes in the hot set, warm set and cold set. In a word, the RHO can simply integrate with intelligent energy perception that achieves efficient energy control under various load conditions. Fog nodes have multiple energy states which consume different levels of energy. S0, S3 and S4 represent active state, sleep state and hibernate state respectively. The energy consumption of the sleep state is close to that of the hibernate state. However, it takes longer time to switch a fog node from the hibernate state to the active state. As a result, the energy efficiency of sleep state is better than that of the hibernate state. The three energy states correspond to hot set, warm set and cold set, respectively. First, the fog nodes in the hot set work in active state with the goal of guaranteeing QoS requirements. Second, the fog nodes in the warm set work in sleep state with the goal of balancing QoS requirements and energy consumption. Third, the fog nodes in the cold set work in hibernate state with the goal reducing energy consumption. By dynamically switching the fog nodes into different sets, we can guarantee QoS requirements and achieve intelligent energy perception.

**Figure 4:**
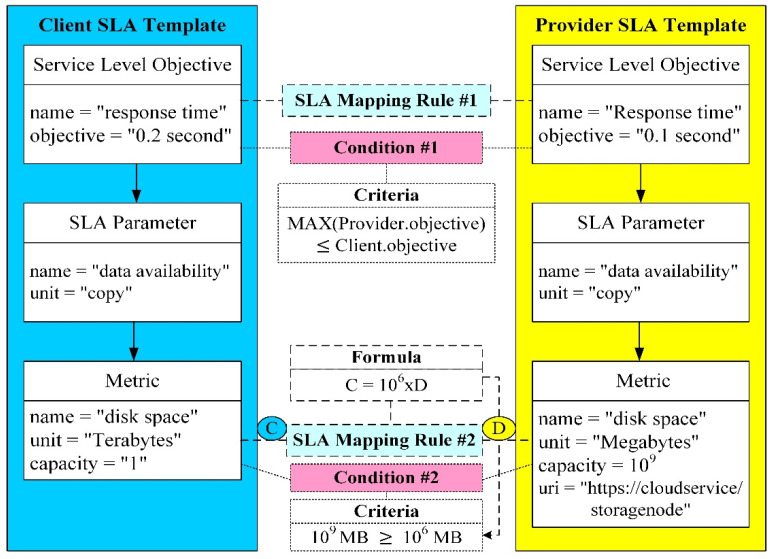
SLA mapping mechanism.

Apart from intelligent energy perception, the fog controllers are responsible for the SLA planning. WSLA is generally used to describe the relationships between the parties, service definition and obligations in the SLA. There are three main factors including metrics, SLA parameters and functions because they are transparent to different service providers. Besides, the other factors can be set at default settings, such as supporting party and service level objectives. Consequently, we complete the design of an SLA template. There are no unified syntax definitions for the SLA template. How to bridge the SLA templates with different syntax but representing the same semantics becomes the main issue. In the past, many studies only considered the mapping between low-level metrics and high-level SLAs. Therefore, we propose a complete and practical SLA mapping mechanism as depicted in Figure 4. To efficiently bridge the differences between service providers and clients, there are two SLA mapping rules and two conditions in the SLA mapping mechanism. The first rule is used to resolve the response time of the service level objectives. The second rule is more complicated because it has to establish formulas that map different units of metrics. After completing the SLA mapping rules, we further construct the required conditions related to the service level objectives and metrics, respectively. Furthermore, we establish the criteria to judge whether the conditions of the SLA contract are satisfied. We utilize the character-based similarity method because the SLA templates usually consist of short strings with less changing syntax. However, the character-based similarity method cannot deal with strings with large syntax differences but they represent the same meaning of semantics. Therefore, we extract the successful mapping information from the mapping repository and further combine with a case-based reasoning method. As a result, we can efficiently identify the SLA templates with the same semantics. As we mentioned previously, we use intelligent energy perception to adjust the fog nodes into a hot set, warm set and cold set that contributes to efficient energy saving. Finally, the RHO is in charge of dynamical replica management. Once it is possible to violate the SLA contract, such as data availability and delay, more fog nodes will be transferred into the hot set by intelligent energy perception.

Next, we introduce the RHO scheme. First, we use Equation (1) to estimate the average load of fog node *i* at the beginning of *k*th time interval, which is denoted by $load_{i,k}^{}$. Similarly, The $load_{i,k-1}^{}$ denotes the average load of fog node *i* at the beginning of (*k* − 1)th time interval. $load_{i,k-1}^{current}$ denotes the current load of fog node *i* during (*k* − 1)th and *k*th time interval. Finally, $k_{a}$ is a parameter whose functionality is used to prevent the estimate of $load_{i,k}^{}$ from being affected by short-term or long-term load variations. $T_{d}$ denotes the duration of each time interval.

(1) $$load_{i,k}^{}=k_{a}\times load_{i,k-1}^{}+\left(1-k_{a}\right)\times load_{i,k-1}^{current}$$

The $load_{i,k-1}^{current}$ is the sum of $hotspot\_load_{i,k-1}^{current}$ and $nonhotspot\_load_{i,k-1}^{current}$, which represent the current loads of hotspot services and non-hotspot services in fog node *i* during (*k*−1)th and *k*th time interval respectively. We adopt the same formula to estimate the $hotspot\_load_{i,k}^{}$ and $\mathrm{non}hotspot\_load_{i,k}^{}$ where $hotspot\_load_{i,k}^{}$ and $\mathrm{non}hotspot\_load_{i,k}^{}$ denote the average load of hotspot services and non-hotspot services in fog node *i* at the beginning of *k*th time interval, respectively. Next, we present the definitions of a hotspot service and a non-hotspot service. $A_{i,k}^{j}$ denotes the amount of arriving requests for fog node *i* with service *j* during (*k*−1)th and *k*th time interval. If $A_{i,k}^{j}$ exceeds a hotspot threshold $hotspot_{th}$, then service *j* is classified as hotspot service; otherwise, service *j* is classified as non-hotspot service. If $hotspot_{load}{}_{i,k}^{}/\left(nonhotspot\_load_{i,k}^{}+hotspot_{load}{}_{i,k}^{}\right)\geq hotspot_{th}$, then fog node *i* is identified as a hotspot node. Otherwise, the fog node *i* is identified as a non-hotspot node. To reduce frequent updates of load variations, we classify the loads into different levels. $weight_{p}$ denote the weight of load level *p*. N is the number of levels. $k_{b}$ and $k_{c}$ determine the level structure. *N* must be a multiple of $k_{c}$ and *C* denotes the maximum capacity of request processing.

(2) $$weight_{p}=\{\begin{array}{c} \begin{array}{cc} \;k_{b}^{p/k_{c}-N/k_{c}}/k_{c} & \;k_{c}+1\leq p\leq N \end{array}\\ \begin{array}{cc} k_{b}^{1-N/k_{c}}/k_{c} & \;\;1\leq p<k_{c}+1 \end{array} \end{array}$$

Next, we use Equation (3) to transfer the loads into corresponding load levels.

(3) $$level_{i,k}^{}=j\;\;\;\;where\;C\times \overset{j}{\underset{p=1}{\sum }}weight_{p}/\overset{N}{\underset{p=1}{\sum }}weight_{p}\leq load_{i,k}^{}<C\times \overset{j+1}{\underset{p=1}{\sum }}weight_{p}/\overset{N}{\underset{p=1}{\sum }}weight_{p}$$

To balance real-time updating and communication overheads, we propose a two-layer updating mechanism using Equation (4). $update_{i,k}^{}$ denotes the information at which a fog node updates its load conditions. $level_{th}$ denotes a threshold of load level and $load_{th}$ denotes a threshold of load variation. If $\left|level_{i,k}^{}-level_{i,k-1}^{}\right|\geq level_{th}$, then fog node *i* broadcasts its load level accompanied by the residual number of replicas to other nodes. Otherwise, we further check the fine load variations. If $|load_{i,k}^{}-load_{i,k-1}^{}|\geq load_{th}$, then the mechanism updates current average load. If $\left|level_{i,k}^{}-level_{i,k-1}^{}\right|<level_{th}\;and\;|load_{i,k}^{}-load_{i,k-1}^{}|<load_{th}$, then no updating is needed. Accordingly, the two-layer updating mechanism improves communication overheads while keeping real-time updating because it dynamically updates coarse load levels or fine average loads based on load conditions.

(4) $$update_{i,k}^{}=\{\begin{array}{c} level_{i,k}^{}\;\;\;\;\;\;\;\;\;\;\;\;\;\;\;\;\;\;\;\;\;\;\;\;\;\;\;\;\;\;\;\;\;\;\;\;\;\left|level_{i,k}^{}-level_{i,k-1}^{}\right|\geq level_{th}\\ load_{i,k}^{}\;\;\;\;\;\;\;\left|level_{i,k}^{}-level_{i,k-1}^{}\right|<level_{th}\;and\;|load_{i,k}^{}-load_{i,k-1}^{}|\geq load_{th} \end{array}\;\;\;\;$$

Next, those fog nodes with minimum replicas of total hotspot service are sequentially selected and they are used to allocate new replicas based on Equation (5). $r_{default}$ denotes the number of replicas of hotspot service. To simplify the replica estimation, we adopt a static number of replicas. ${\mathrm{node}}_{i}^{set}$ denotes the set of non-hotspot fog nodes that already have a replica of the same hotspot service for fog node *i*. ${\mathrm{node}}_{i}^{add}$ denotes a set of new fog nodes added to ${\mathrm{node}}_{i}^{set}$. min(x) is the smallest element in x. In brief, RHO distributes replicas based on the number of replicas of total hotspot service residing in each fog node. A new replica represents that a certain of requests be dispatched to other fog nodes. Thus, RHO greatly improves load imbalances.

(5) $${\mathrm{node}}_{i}^{add}=\overset{m=r_{default}}{\underset{m=1}{\sum }}\{h|\min\left(replica_{h}^{}\right)\;where\;h\notin {\mathrm{node}}_{i}^{set}\}\;$$

In RHO, the master fog nodes are responsible for scheduling the arriving requests to related slave fog nodes with the required services. The master fog node selects the fog node with the minimum load level based on Equation (6).

(6) $$dispatch_{i,k}=\{h|\min\left(level_{h,k}^{}\right)\;where\;h\in {\mathrm{node}}_{i}^{set}\}$$

## 4. Simulation Results

We developed a software simulator to perform all simulations, which was designed to generate and process requests. We considered the application scenarios of the augmented reality (AR) service which was deployed in the fog nodes while using container technology. Unlike a virtual machine, the container was an efficient virtualization because it used a host operating system, shared libraries and related resources. In other words, the container only requires application-specific binaries and container libraries that apparently speed up the service deployment. As a result, it required a fraction of a second to launch a container. Therefore, our study is feasible for such application scenarios. We used computer simulations to compare the fairness of the RHO scheme with ripple load balancing (RLB) and optimal load-balancing (OLB) schemes. In the simulations, we assumed that the system architecture consisted of one fog network where 64 fog nodes and one fog controller existed. The users accessed the service deployed in the fog nodes and hence various requests were generated. We used ON–OFF models to simulate the request behaviors of hotspot service. Furthermore, we used ON–OFF models to simulate the load variations of non-hotspot service. Both RHO and RLB schemes work with distributed control but the OLB scheme works with centralized control. In OLB, we assumed that the fog controller always obtains the real-time load conditions of fog nodes. Furthermore, each fog node maintains a replica of the each resided service. As a result, the fog controller can forward the requests to the fog node with the required service while having the lowest load. The OLB is the most impractical algorithm. First, the OLB has the highest communication overheads because it requires real-time fog node information. Without this, it greatly degrades fairness performance. Second, the fog controller might become the performance bottleneck because it has to completely deal with all arriving requests. Third, to provide real-time load conditions of fog nodes is too difficult to implement. Fourth, it needs to duplicate the same service to all fog nodes that consumes a lot of resources such as storage, computing and bandwidth. In all experiments, the OLB worked as a benchmark of the fairness performance. RLB maintained a fixed number of replicas for resided services. In other words, RLB belongs to the static strategy. 

**Figure 5:**
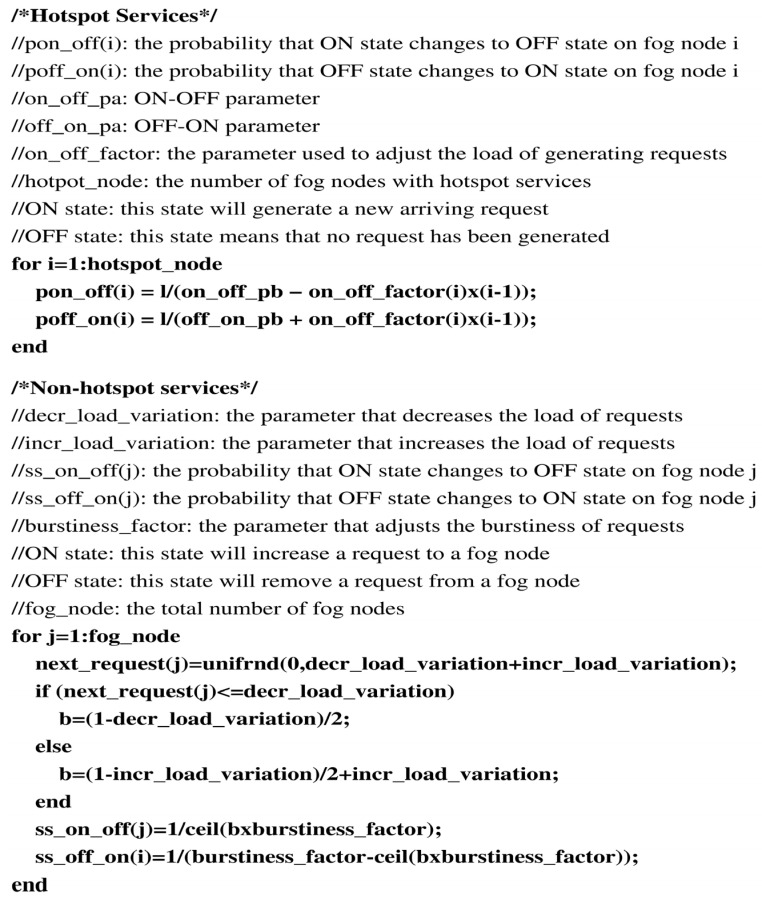
Request-generating models.

Unless otherwise specified, we used the following parameter settings in all experiments. The RHO’s parameters were set as follows: $k_{a}=0.3$, $k_{b}=2$, $k_{c}=2$, N=16, $level_{th}=1$, $load_{th}=250$ and $T_{d}=15$ min. Furthermore, all fog nodes possessed the same request processing capacity, that is *C* =25,000. We considered 64 fog nodes in our simulations. Finally, there were 10 replicas for a hotspot service. The RLB’s parameters were set as follows: $k_{a}=0.8$, $k_{b}=0.01$. The default number of requests residing in each fog node was between 7500 and 12,500, randomly assigned in each experiment. The total duration of each experiment was 20 h. The parameter definitions of the request generating models are depicted in Figure 5. The OLB adopted the same scenarios as RLB and RHO. Besides, the OLB had to deploy the same services to all fog nodes.

**Figure 6:**
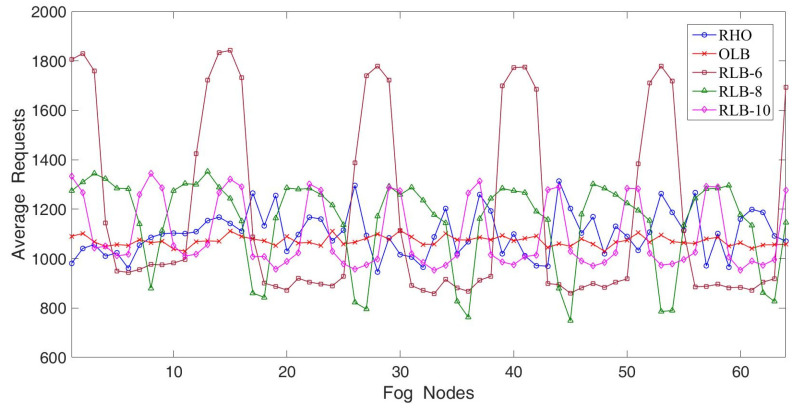
The average requests where sixteen fog nodes have high loads of hotspot services.

In the first experiment, we considered 16 fog nodes having high loads of hotspot services. The experimental results are illustrated in Figure 6. The parameter settings were as follows; on_off_pb = 160, off_on_pb = 40, on_off_factor = 2, incr_load_variation = 0.1, decr_load_variation = 0.58 and burstiness_factor = 200. Apparently, fog node 1 had the largest number of arriving requests, while fog node 16 had the lowest number of arriving requests. In other words, fog node 1 to fog node 16 were heavily congested. The OLB achieved the best fairness because all fog nodes had almost the same and stable number of average requests. The OLB was capable of redirecting requests in time to whichever fog node had the lowest number of residing requests. The reason is that each fog node keeps replicas of hotspot services. To make the OLB work well, each fog node has to maintain a replica of residing services so that real-time duplication is needed. Therefore, the OLB had the lowest storage utilization. In addition, each fog node had to timely and frequently update their load information to the fog controller and hence the OLB had extremely high communication overheads. RLB-6 denotes that RLB maintains six replicas for residing services, RLB-8 denotes that RLB maintains eight replicas for residing services, and so on. When the number of replicas increased from 6 to 10, RLB achieved better fairness because fog nodes with hotspot service were able to redirect more requests to other fog nodes which only had non-hotspot services. The RHO’s fairness approached that of OLB but was better than that of RLB-10. This is mainly because the RHO dynamically dispatched arriving requests to the fog nodes with lower loads. Despite requiring fewer replicas, the RHO tended to approach the best fairness of the OLB, and apparently outperformed the RLB scheme.

**Figure 7:**
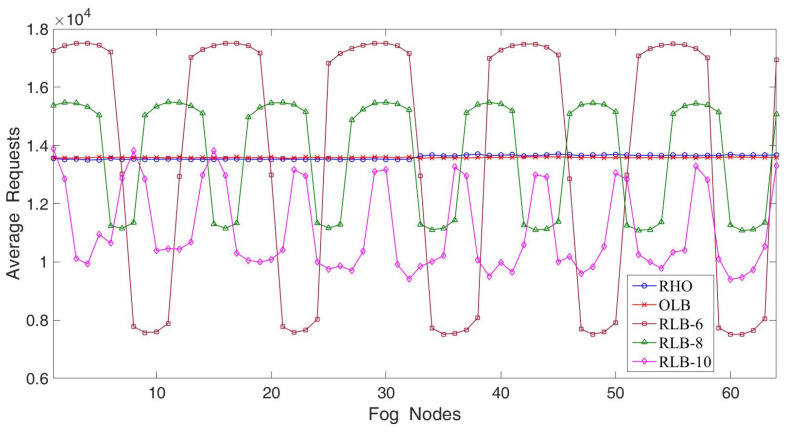
The average requests where thirty-two fog nodes have high loads of hotspot services.

In the second experiment, we considered the effect of the increment of fog nodes with hotspot services. The number of fog nodes increased from 16 to 32 and therefore the overall load variations increased as compared with the first experiment. The simulation results are illustrated in Figure 7. The other parameter settings were the same values as the first experiment. Repeatedly, the OLB demonstrated the best fairness. In evaluating the fairness of RLB-6, RLB-8 and RLB-10, we found that increasing replicas had less improvement on fairness. The overall throughput of RLB-10 apparently decreased because RLB was incapable of allocating replicas effectively. Therefore, many arriving requests were discarded due to limited request processing capacity. The fairness of the RHO was close to that of the OLB because more fog nodes with non-hotspot services had replicas of the fog nodes with hotspot services. With that, RHO greatly enhanced request dispatches. As a result, the performance of the RHO still approximated to that of the OLB. In addition, it was much better than that of the RLB. 

**Figure 8:**
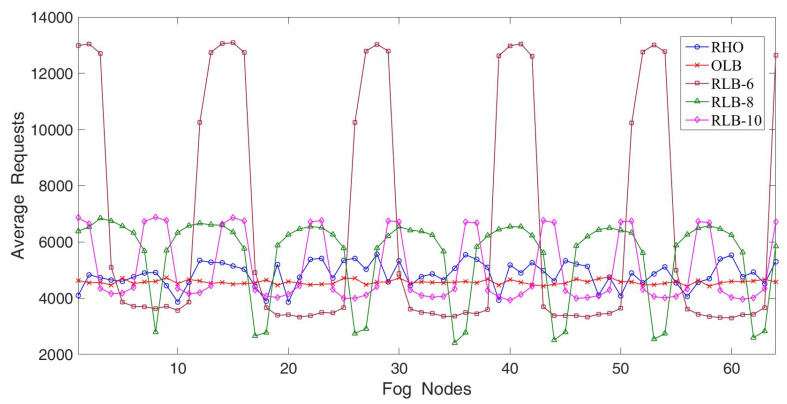
The average requests where sixteen fog nodes have different load conditions.

In the third experiment, we studied the effect on fairness when 16 fog nodes had different load conditions. The parameter settings including the default number of requests residing in each node was between 5000 and 20,000 and incr_load_variation was set at 0.2. The simulation results are illustrated in Figure 8. Due to increasing load variations, the RLB demonstrated relatively poor fairness as compared with the first experiment. However, the RHO still demonstrated excellent fairness and approached the performance of OLB. From Figure 6, Figure 7 and Figure 8, the RHO apparently outperformed the RLB and came close to the OLB under various scenarios, such as the number of fog nodes with hotspot services and load variations. In a word, the simplicity of the RHO was similar to that of the RLB but the RHO achieved excellent fairness close to the OLB.

## 5. Conclusions

In this paper, we proposed an integrated resource management for fog networks inclusive of intelligent energy perception, service level agreement planning and replication-based hotspot offload. First, fog nodes were dynamically classified into a hot set, warm set or cold set in terms of load conditions or a pre-defined number of fog nodes in each set. The fog nodes in the hot set are responsible for guaranteeing QoS and the fog nodes in the cold set are maintained at a low-energy state to save energy consumption. Moreover, the fog nodes in the warm set are used to balance the QoS requirements and energy consumption. Secondly, we described the relationships between resource metrics and SLA parameters. Accordingly, we proposed an SLA mapping mechanism which efficiently identified the SLA elements with the same semantics. Finally, we proposed the replication-based hotspot offload scheme which could be easily integrated with intelligent energy perception and service level agreement planning. The RHO scheme is easy to implement and it achieves excellent fairness. In addition, it has limited communication overheads and requires a limited number of replicas. The simulation results demonstrated that the fairness of the RHO outperformed that of the RLB and approached that of the OLB. In the future, we will further study ways to contribute to improving the practicality and efficiency for integrated resource management. First of all, we will design a transmission control protocol (TCP) latency model so that the study can approach practical situations. Furthermore, we will design an energy model that carries out the study for in depth evaluation of the energy consumption of the intelligent energy perception. In the end, we will consider the mobile environments and therefore the study can be applied to analyze more wide and complicated fog networks.

## Data Availability

Not applicable.
